# Detecting *erm*-Mediated Inducible Macrolide–Lincosamide–Streptogramin B Resistance in Anaerobic Clinical Isolates

**DOI:** 10.3390/antibiotics15040360

**Published:** 2026-04-01

**Authors:** Fabio Daniel Thalmann, Claudio Neidhöfer, Pascal Schläpfer, Christopher Field, Karoline Leuzinger, Claudia Lang, Peter M. Keller

**Affiliations:** 1 Division of Clinical Bacteriology and Mycology, University Hospital Basel, 4031 Basel, Switzerland; 2 Institute of Medical Genetics and Pathology, University Hospital Basel, 4031 Basel, Switzerland; 3 Center for Infectious Disease Diagnostics, University Hospital Basel, 4031 Basel, Switzerland; 4 Laboratory Medicine, University Hospital Basel, 4031 Basel, Switzerland; 5 Clinical Virology, University Hospital Basel, 4031 Basel, Switzerland

**Keywords:** anaerobes, erythromycin, clindamycin, *erm*, inducible resistance, bacterial WGS, D-test

## Abstract

**Background**: Inducible macrolide–lincosamide–streptogramin B (iMLSB) resistance is well defined in Gram-positive aerobes but remains poorly characterized in anaerobes, where standardized detection strategies are lacking. Following withdrawal of EUCAST guidance to infer clindamycin resistance from erythromycin resistance in *Peptostreptococcus* and *Bacteroides* spp. because of inconsistent species-specific performance, a diagnostic gap persists. **Methods**: We therefore assessed the accuracy of the D-test for detecting iMLSB resistance in anaerobes by correlating phenotypic results with whole-genome sequencing data. Fifty clinical anaerobic isolates, including *Finegoldia magna*, *Peptostreptococcus anaerobius*, and *Bacteroides* spp., were included in the analysis. Antimicrobial susceptibility testing was performed using gradient diffusion to determine minimum inhibitory concentrations of erythromycin and clindamycin, complemented by D-test analysis for phenotypic detection of inducible resistance. Whole-genome sequencing was undertaken to identify *erm* genes encoding ribosomal methyltransferases associated with the iMLSB phenotype. **Results**: Among the 50 isolates, *erm* genes were detected in 16 strains (32.0%). The prevalence of *erm* positivity was highest among Gram-positive cocci (50%), followed by Gram-positive rods (35.3%) and Gram-negative rods (11.8%). Five *erm*-positive isolates exhibited a characteristic D-shaped growth pattern, with high erythromycin MICs (>256 mg/L) and low clindamycin MICs (≤2 mg/L), consistent with an inducible iMLSB phenotype, whereas the remaining eleven demonstrated constitutive resistance. **Conclusions**: The D-test accurately identified inducible iMLSB resistance among Gram-positive anaerobic cocci and, if confirmed in larger studies, could form the basis of an accessible and pragmatic screening strategy for this subgroup. Integration of molecular analyses seems essential for the evidence-based refinement of diagnostic algorithms, particularly in the absence of robust, species-specific guidance.

## 1. Introduction

Inducible resistance to macrolide, lincosamide, and streptogramin B antibiotics (iMLSB) represents a clinically relevant challenge in the management of Gram-positive infections, particularly those caused by staphylococci and streptococci [1,2]. This phenotype, characterized by resistance that becomes apparent only upon antibiotic exposure, complicates therapeutic decision-making and underscores the importance of reliable detection methods [3,4]. In contrast to aerobic pathogens, the prevalence, molecular basis, and optimal laboratory detection of iMLSB resistance in anaerobic bacteria remain insufficiently defined, and dedicated studies are scarce [3].

In our laboratory, antimicrobial susceptibility interpretation previously followed EUCAST expert rules Version 2.0 [5], whereby clindamycin resistance in *Peptostreptococcus* spp. and *Bacteroides* spp. was inferred from erythromycin resistance determined by gradient testing. Although pragmatic, this approach was later withdrawn in subsequent EUCAST revisions because of inconsistent, species-dependent performance, leaving uncertainty regarding appropriate detection strategies for iMLSB resistance in anaerobes.

Macrolides, lincosamides, and streptogramin B antibiotics act by binding to the 23S rRNA of the 50S ribosomal subunit, obstructing the peptide exit tunnel and thereby inhibiting protein synthesis [6]. Resistance to MLSB agents arises predominantly through three mechanisms: target-site modification via methylation of the 23S rRNA, typically mediated by *erm* genes and expressed either constitutively (cMLSB) or inducibly (iMLSB); active efflux or reduced uptake; and enzymatic inactivation of the antibiotic molecule [7]. In routine disk diffusion testing with erythromycin and clindamycin, these mechanisms give rise to distinct phenotypic patterns, including the characteristic D-shaped zone of inhibition associated with inducible resistance [8].

Against this background, we aimed to evaluate the D-test as a tool for detecting iMLSB resistance in anaerobic bacteria. By correlating erythromycin and clindamycin minimum inhibitory concentrations and D-test phenotypes with whole-genome sequencing data identifying *erm* determinants, we sought to clarify the diagnostic performance of phenotypic testing and to inform the optimization of susceptibility testing algorithms for clinically relevant anaerobic pathogens.

## 2. Results

### 2.1. Species Distribution

During the study period, 56 anaerobic strains submitted for antimicrobial susceptibility testing were analyzed. Six isolates were excluded due to inconsistencies in retrievability, missing data or species identification. The remaining 50 anaerobic clinical isolates were analyzed (see Table 1), with *Cutibacterium acnes* (*n* = 10), *Finegoldia magna* (*n* = 7), and *Bacteroides fragilis* (*n* = 7) representing the most frequently recovered species. Isolates were further categorized into anaerobic subgroups: Gram-positive cocci (gpc), Gram-positive rods (gpr), and Gram-negative rods (gnr).

### 2.2. Detection of erm-Genes

Sixteen (32.0%) of the 50 isolates carried an *erm* gene. Distribution of *erm* genes was as follows: *erm*(A) (8/16, 50.0%), *erm*(X) (4/16, 25.0%), *erm*(B) (2/16, 12.5%), and *erm*(F) (2/16, 12.5%). Both *erm*(F)-positive isolates were Gram-negative rods, whereas *erm*(A), *erm*(B), and *erm*(X) were found in Gram-positive species. *erm*(A) was exclusively detected in Gram-positive cocci, *erm*(B) and *erm*(X) only in Gram-positive rods.

### 2.3. Correlation of erm-Genes to Erythromycin and Clindamycin MICs

Among the 50 isolates, 20 (40.0%) showed elevated MIC values to erythromycin, qualifying the isolates as suspicious for MLSB resistance (see Table 2). Ten of these isolates were simultaneously resistant to clindamycin, indicating constitutive MLSB (cMLSB) resistance. Whole-genome sequencing (WGS) revealed that all 10 (100%) isolates resistant to both clindamycin and erythromycin carried an *erm* gene (*erm*(A), *erm*(B), *erm*(F), or *erm*(X)). Of the remaining ten clindamycin-susceptible (and thus potentially iMLSB positive) isolates, six (60%) carried an *erm*(A) gene. All these six isolates belonged to the subgroup of Gram-positive cocci with *Finegoldia magna* making up 5/6 of these isolates.

### 2.4. D-Test Results

**Figure 1:**
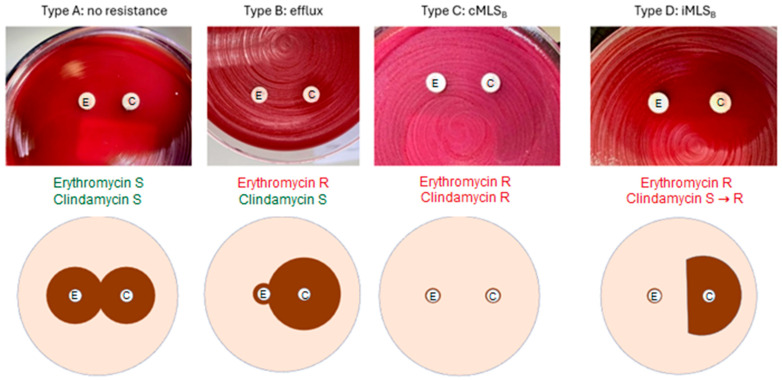
Phenotypic D-test patterns corresponding to different resistance mechanisms. Phenotypes A–D illustrate representative inhibition patterns. Type A: no resistance (susceptible to erythromycin and clindamycin). Type B: phenotype compatible with efflux-mediated resistance (macrolide-resistant, clindamycin-susceptible). Type C: constitutive MLSB resistance (no inhibition zone: growth around both disks). Type D: inducible MLSB resistance (D-shaped blunting of the clindamycin inhibition zone adjacent to the erythromycin disk). Schematic diagrams below show simplified inhibition zones corresponding to each phenotype. Accordingly, type D corresponds to a D-test-positive result, whereas types A–C represent D-test-negative results.

D-test phenotypes and corresponding genotypic findings are summarized in Figure 1 and Table 3. Twenty-two isolates exhibited full susceptibility to erythromycin and clindamycin (type A phenotype); none harbored *erm* genes. Six isolates were consistent with a phenotype compatible with efflux-mediated resistance (type B pattern); all were *erm*-negative and remained susceptible to clindamycin, although four showed high-level erythromycin resistance (MIC > 256 mg/L). A constitutive MLSB phenotype (type C) was observed in eleven isolates, ten of which carried *erm* determinants and demonstrated high-level resistance to both erythromycin and clindamycin (MICs > 256 mg/L).

One *Finegoldia magna* isolate was *erm*(A)-positive and resistant to erythromycin (>256 mg/L) but retained clindamycin susceptibility (MIC 2 mg/L). An inducible MLSB phenotype (type D) was identified in five *erm*(A)-positive Gram-positive cocci. All were resistant to erythromycin yet susceptible to clindamycin, with MICs ranging from 0.25 to 1.5 mg/L. For six isolates, phenotypic classification was discordant between the two independent reviewers, with interpretations alternating between type A and type B. All belonged to the Gram-negative rod group, exhibited clindamycin and erythromycin MICs below established resistance thresholds, and lacked detectable *erm* genes.

Minor discrepancies were observed between D-test and MIC interpretation. The first interpretation of the growth patterns was made by biomedical analysts, specialized in detecting the type D growth pattern (See Figure 1), to search for MLSB suspicious isolates. A second interpretation by trained clinical microbiologists aimed to assign the growth patterns to type A to D. Six tests (12%) were read differently by biomedical analysts and clinical microbiologists.

Detailed diagnostic performance metrics for clindamycin resistance prediction across phenotypic and MIC-based approaches (Table A1) are provided in the Appendix A.

## 3. Discussion

This study provides a first systematic assessment of MLSB resistance mechanisms among anaerobic clinical isolates recovered at a tertiary-care center and addresses an area in which diagnostic guidance remains limited. As antimicrobial selection pressure continues to drive adaptive resistance, a precise understanding of underlying mechanisms is essential to preserve therapeutic options. In anaerobes, however, resistance detection is frequently complicated by slow growth, limited standardization of testing strategies, and reduced routine laboratory familiarity, all of which may delay recognition of clinically relevant phenotypes [3,9,10].

Clindamycin remains an established component of treatment recommendations for anaerobic infections and retains practical advantages, including oral availability and low cost. Nevertheless, rising resistance rates have been reported, raising concern about unrecognized inducible mechanisms [1,2,11,12]. In this context, our data suggest that the D-test may offer a pragmatic adjunct to conventional MIC-based assessment for the identification of inducible MLSB resistance, particularly in selected subgroups of anaerobes.

Consistent with prior literature [4,7,8,13,14,15], *erm* determinants—most notably *erm*(A)—were strongly associated with inducible phenotypes. The absence of detectable *erm* genes in a subset of resistant isolates indicates that alternative mechanisms, potentially including efflux-mediated pathways, may contribute. Although the sample size limits statistical power, the inclusion of consecutively collected routine isolates ensured representation of clinically relevant species.

The observed interobserver variability in D-test interpretation underscores the need for clear implementation standards to ensure reproducibility and emphasizes the need for training and refresher sessions to standardize interpretation. The incremental diagnostic yield beyond EUCAST-guided MIC categorization was modest overall, as most isolates with a positive D-test were already classified as resistant. However, in Gram-positive anaerobic cocci, the assay provided meaningful discrimination between inducible and constitutive resistance phenotypes.

Taken together, the combined application of MIC testing and targeted D-test assessment represents a rational strategy for detecting inducible MLSB resistance in anaerobes. In the absence of formal guidance, one possible approach could be to report clindamycin as resistant or to include an interpretive comment in Gram-positive anaerobic cocci with a positive D-test despite a susceptible MIC. In our cohort, the added value of D-testing was largely confined to erythromycin-resistant, clindamycin-susceptible Gram-positive cocci, where inducible resistance may escape MIC-based classification. Given the limited number of isolates analyzed, particularly the small number of inducible (type D) phenotypes, these findings should be considered preliminary and seek confirmation.

Larger, prospective studies are needed to define its clinical impact and to refine diagnostic algorithms in this understudied group of pathogens. In parallel, systematic whole-genome sequencing of routine clinical isolates, integrated with phenotypic susceptibility data, enables the delineation of resistance and virulence determinants in a manner that extends beyond individual cases [16,17,18,19], generating knowledge that remains applicable even when sequencing is not performed and thereby informing future therapeutic and diagnostic decision-making.

## 4. Materials and Methods

This study represents a secondary analysis of data from a prospective strain collection of routine clinical isolates obtained from patients with anaerobic bacterial infections. A total of 56 anaerobic bacterial strains were collected between August and December 2023 from blood cultures, swabs, and implant materials.

### 4.1. Culture and Identification

Microscopy, aerobic, and anaerobic culture of the specimens were performed following standard microbiological procedures. Anaerobic cultures were incubated and handled in an anaerobic workstation (Whitley A95, Don Whitley Scientific Ltd., Bingley, UK). Species identification was based on colony morphology and confirmed by matrix-assisted laser desorption/ionization time-of-flight mass spectrometry (MALDI-ToF MS; Bruker Daltonics, Bremen, Germany). Isolates with inconclusive MALDI-ToF MS results underwent 16S rRNA gene PCR and sequencing as described by [20].

### 4.2. In Vitro Antimicrobial Susceptibility Testing

Minimum inhibitory concentrations (MICs) were determined by gradient diffusion using commercial strips (Liofilchem, Roseto degli Abruzzi, Italy). Interpretation followed EUCAST v15.0 (2025) and CLSI M100, 35th edition (2025) criteria. Species-specific breakpoints for clindamycin were applied as recommended: *Bacteroides* spp. (susceptible ≤ 4 mg/L), *Prevotella* spp., *Fusobacterium necrophorum* and *Cutibacterium acnes* (≤0.25 mg/L), and other anaerobic species (≤2 mg/L). For erythromycin, isolates were considered suspicious for MLSB-associated resistance at MICs ≥ 48 mg/L in Gram-negative anaerobes, including *Bacteroides* spp., and ≥12 mg/L in Gram-positive anaerobes. These thresholds correspond to the EUCAST expert-rule cutoffs (>8 mg/L and >32 mg/L, respectively) but were rounded to the next higher dilution steps used in gradient strip MIC testing to facilitate routine interpretation [5].

### 4.3. D-Test for Inducible Clindamycin Resistance

D-tests were performed on Brucella agar using erythromycin (15 µg) and clindamycin (2 µg) disks positioned 12–20 mm apart, as previously described [21]. Plates were incubated under anaerobic conditions at 35 °C and examined after 24–48 h; incubation was extended to up to 72 h when required to allow adequate growth or clearer expression of inhibition zone morphology. Results were independently interpreted and photographically documented by two biomedical scientists. In cases of discordant, the result was reviewed by a third reader, a clinical microbiologist, whose assessment served as the final interpretation.

Four phenotypic patterns were defined as depicted in Figure 1: type A, absence of growth around both disks, indicating full susceptibility; type B, growth confined to the erythromycin disk, consistent with an efflux-mediated phenotype; type C, growth surrounding both disks (no inhibition zone), reflecting constitutive MLSB resistance; and type D, blunting of the clindamycin inhibition zone adjacent to the erythromycin disk, indicative of inducible MLSB resistance. The D-test therefore permits phenotypic discrimination between constitutive, inducible, and efflux-mediated resistance mechanisms, with direct implications for antimicrobial selection [22].

### 4.4. Whole Genome Sequencing

For each isolate, an antimicrobial susceptibility profile was established, including disk diffusion and D-test assays, and whole genome sequencing (WGS) for the detection of *erm* genes associated with iMLSB resistance. The presence of *erm* genes was compared with phenotypic D-test results for concordance.

Genomic DNA was extracted using the EZ1 DNA Tissue Kit and EZ1 Advanced XL workstation (QIAGEN, Hilden, Germany) according to the manufacturer’s protocol. Libraries were prepared using the Illumina DNA Prep kit and sequenced on a NextSeq 500 platform (Illumina, San Diego, CA, USA) with 2 × 150 bp reads. Genome assembly was performed using Unicycler (v0.3.0b), and annotation and gene analysis were conducted in Ridom SeqSphere+ (v7.7.5) (Ridom, Münster, Germany). Genome data of the isolates of this study is accessible at ENA under BioProject number PRJEB108527.

### 4.5. Statistics

Sensitivity, specificity, positive predictive value (PPV), and negative predictive value (NPV) were calculated using standard contingency table analysis.

### 4.6. Ethics

This study used bacterial isolates only, without any clinical data or identifiable patient information. It is therefore exempt from approval under the Swiss Human Research Act.

## 5. Conclusions

Anaerobic antimicrobial resistance represents an increasingly relevant clinical concern. Inducible macrolide–lincosamide resistance mediated by *erm* genes may compromise established treatment options, including clindamycin. Since the revision of EUCAST expert rules in 2017, however, no standardized strategy for the detection of inducible MLSB resistance in anaerobes has been defined. In our cohort, the diagnostic contribution of the D-test appeared limited in anaerobic Gram-positive and Gram-negative rods but provided additional information in Gram-positive cocci, where inducible phenotypes were associated with the presence of erm determinants. These observations suggest that, in this subgroup, the D-test could represent a pragmatic and low-cost adjunct to routine susceptibility testing.

## Figures and Tables

**Table 1:** Distribution of anaerobic isolates and presence of *erm* genes.

Bacterial Group/Species	No. of Isolates (%)	*erm*-Positive Isolates (n)
**Gram-positive cocci**	**16 (32.0)**	**8**
*Anaerococcus* spp.	2 (4.0)	1
*Finegoldia magna*	7 (14.0)	6
*Helcococcus kunzii*	1 (2.0)	1
*Lancefieldella* spp.	2 (4.0)	–
*Parvimonas micra*	1 (2.0)	–
*Peptoniphilus* spp.	2 (4.0)	–
*Staphylococcus saccharolyticus*	1 (2.0)	–
**Gram-positive rods**	**17 (34.0)**	**6**
*Clostridium innocuum* ^1^	2 (4.0)	2
*Cutibacterium acnes*	10 (20.0)	1
*Cutibacterium avidum* ^1^	2 (4.0)	1
*Cutibacterium granulosum*	1 (2.0)	1
*Schaalia turicensis*	1 (2.0)	–
*Winkia neuii*	1 (2.0)	1
**Gram-negative rods**	**17 (34.0)**	**2**
*Bacteroides caccae*	1 (2.0)	–
*Bacteroides fragilis*	7 (14.0)	1
*Fusobacterium necrophorum*	1 (2.0)	–
*Fusobacterium nucleatum*	3 (6.0)	–
*Prevotella bivia*	1 (2.0)	1
*Prevotella histicola*	1 (2.0)	–
*Prevotella melaninogenica*	2 (4.0)	–
*Prevotella oralis*	1 (2.0)	–
**Total**	**50 (100.0)**	**16**

Abbreviations: –, no *erm* detected. ^1^ Including one isolate with lower ANI (average nucleotide identity) score, potentially novel species.

**Table 2:** Phenotypic and genotypic analysis of antimicrobial resistance in isolates with potential MLSB resistance.

Species	Bacterial Group	Erythromycin MIC mg/L	Clindamycin MIC mg/L	Clindamycin Interpretation	D-Test	Detected *erm* Genes
*Anaerococcus* spp.	gpc	>256	0.38	S	D	*erm*(A)
*Finegoldia magna*	gpc	>256	1.5	S	D	*erm*(A)
*Finegoldia magna*	gpc	>256	1	S	D	*erm*(A)
*Finegoldia magna*	gpc	>256	1	S	D	*erm*(A)
*Finegoldia magna*	gpc	>256	0.25	S	D	*erm*(A)
*Finegoldia magna*	gpc	>256	>256	R	C	*erm*(A)
*Finegoldia magna*	gpc	>256	2	S	C	*erm*(A)
*Helcococcus kunzii*	gpc	>256	>256	R	C	*erm*(A)
*Finegoldia magna*	gpc	>256	0.38	S	B	–
*Clostridium innocuum*	gpr	>256	>256	R	C	*erm*(B)
*Clostridium innocuum*	gpr	>256	>256	R	C	*erm*(B)
*Cutibacterium acnes*	gpr	>256	>256	R	C	*erm*(X)
*Cutibacterium avidum*	gpr	>256	>256	R	C	*erm*(X)
*Cutibacterium granulosum*	gpr	>256	>256	R	C	*erm*(X)
*Winkia neuii*	gpr	>256	>256	R	C	*erm*(X)
*Bacteroides fragilis*	gnr	>256	>256	R	C	*erm*(F)
*Prevotella bivia*	gnr	>256	>256	R	C	*erm*(F)
*Bacteroides fragilis*	gnr	>256	0.38	S	B	–
*Bacteroides fragilis*	gnr	>256	0.25	S	B	–
*Fusobacterium nucleatum*	gnr	>256	0.094	S	B	–

Abbreviations: – no *erm* detected; gpc, Gram-positive cocci; gpr, Gram-positive rods; gnr, Gram-negative rods; S, susceptible (highlighted in green); R, resistant (highlighted in red) (interpretation followed EUCAST v15.0 (2025) criteria).

**Table 3:** Concordance of D-test phenotypes with *erm* gene detection and MIC-based susceptibility interpretation.

Disk-Diffusion Test	No. of Tests (%)	Concordant Results *erm* n, (%)	Concordant Result MIC n, (%)
Type A (no resistance)	22 (44%)	22 (100%)	22 (100%)
Type B (efflux)	6 (12%)	6 (100%)	4 (66.7%)
Type C (cMLS_B_)	11 (22%)	10 (90.9%)	10 (90.9%)
Type D (iMLS_B_)	5 (10%)	5 (100%)	5 (100%)
Type Unclear (A/B)	6 (12%)	6 (100%)	

## Data Availability

All relevant data is included in the article. Genome data of the isolates of this study is accessible at ENA under BioProject number PRJEB108527.

## Appendix A

**Table A1:** Clindamycin resistance prediction.

	Subclass	Erythroycin MIC Threshold	Clindamycin Resistance	D-Test (C or D)
Sensitivity	gpc	100% (8/8)	25% (2/8)	100% (8/8)
Specificity	gpc	87.5% (7/8)	100% (8/8)	100% (8/8)
Accuracy	gpc	93.8% (15/16)	62.5% (10/16)	100% (16/16)
Sensitivity	gpr	100% (6/6)	100% (6/6)	100% (6/6)
Specificity	gpr	100% (11/11)	100% (11/11)	100% (11/11)
Accuracy	gpr	100% (17/17)	100% (17/17)	100% (17/17)
Sensitivity	gnr	100% (2/2)	100% (2/2)	100% (2/2)
Specificity	gnr	80% (12/15)	100% (15/15)	100% (15/15)
Accuracy	gnr	82.4% (14/17)	100% (17/17)	100% (17/17)
Sensitivity	all	100% (16/16)	62% (10/16)	100% (16/16)
Specificity	all	88.2% (30/34)	100% (34/34)	100% (34/34)
Accuracy	all	92.0% (46/50)	88.0% (44/50)	100% 50/50)
PPV	all	80.0% (16/20)	100% (10/10)	100% (16/16)
NPV	all	100% (30/30)	85.0% (34/40)	100% (34/34)
